# The current status of surgical care in the Asia–Pacific region and opportunities for improvement: proceedings

**DOI:** 10.1186/s12919-023-00255-0

**Published:** 2023-07-25

**Authors:** Rennie X. Qin, Zachary G. Fowler, Anusha Jayaram, Makela Stankey, Sangchul Yoon, Elizabeth McLeod, Kee B. Park

**Affiliations:** 1 The Program in Global Surgery and Social Change, the, Department of Global Health and Social Medicine , Harvard Medical School, 641 Huntington Ave, Boston, MA 02115 USA; 2 Tufts University School of Medicine, 145 Harrison Ave, Boston, MA 02111 USA; 3 Keck School of Medicine at the University of Southern California, 1975 Zonal Ave, Los Angeles, CA 90033 USA; 4 Department of Medical Humanities and Social Sciences, College of Medicine, Yonsei University, Seoul, South Korea; 5 Department of Neonatal and Paediatric Surgery, Royal Children’s Hospital, 50 Flemington Rd, Melbourne, VIC 3052 Australia

**Keywords:** Global surgery, Surgical system strengthening, National surgical planning, Asia–Pacific, Health systems

## Abstract

The World Health Assembly resolution 68.15 recognised emergency and essential surgery as a critical component of universal health coverage. The first session of the three-part virtual meeting series on *Strategic Planning to Improve Surgical, Obstetric, Anaesthesia, and Trauma Care in the Asia–Pacific Region* focused on the current status of surgical care and opportunities for improvement. During this session, Ministries of Health and World Health Organization (WHO) Regional Directors shared country- and regional-level progress in surgical system strengthening. The WHO Western Pacific Regional Office (WPRO) has developed an Action Framework for Safe and Affordable Surgery, whilst the WHO South-East Asia Regional Office (SEARO) highlighted their efforts in emergency obstetric care, workforce strengthening, and blood safety. Numerous countries have begun developing and implementing National Surgical, Obstetric, and Anaesthesia Plans (NSOAPs). Participants agreed surgical system strengthening is an integral component of universal health coverage, pandemic preparedness, and overall health system resilience. Participants discussed common challenges, such as the COVID-19 pandemic, climate change, workforce capacity building, and improving access for hard-to-reach populations. They generated and shared common solutions, including strengthening surgical care capacity in first-level hospitals, anaesthesia task-shifting, remote training, and integrating surgical care with public health, preventive care, and emergency preparedness. Moving forward, participants committed to developing and implementing NSOAPs and agreed on the need to raise political awareness, build a broad-based movement, and form intersectoral collaborations.

**Supplementary Information:**

The online version contains supplementary material available at 10.1186/s12919-023-00255-0.

## Introduction

On 18^th^ February 2021, 223 participants from 24 countries convened to discuss the current status and country progress towards improving surgical care in the Asia–Pacific region. This is the first session of a three-part meeting series on *Strategic Planning to Improve Surgical, Obstetric, Anaesthesia, and Trauma Care in the Asia–Pacific Region*. Following framing statements on the history and the current status of global surgery, two World Health Organization (WHO) regional directors and five Ministries of Health shared regional- and country-level perspectives and progress in surgical system strengthening. Table 1 summarises each country’s progress in developing National Surgical, Obstetric, and Anaesthesia Plans (NSOAPs) and identified challenges and solutions. Table 2 summarises discussion themes by stakeholder. The session was moderated by Kee Park, Lecturer on Global Health and Social Medicine at Harvard Medical School (HMS), and Elizabeth McLeod, paediatric surgeon at the Royal Children’s Hospital, Australia and Fellow of the Royal Australasian College of Surgeons (RACS).

**Table 1:** Country progress in surgical system strengthening [1, 2]

Country	Population (2021)	GDP per capita (2021)	CHE (% GDP) (2019)	LE (2020)	NSOAP stage	Achievements to date	Challenges	Proposed Solutions & Next Steps
Tonga	106,760	4,624 (2020)	4.98	70.9	Drafting	- Secured political commitment, educated political leaders - Conducted baseline situational analysis - Stakeholder consultation workshop - Drafted the NSOAP	- Education & training - Transportation limitations	- Launch and implement its NSOAP
Cook Islands	17,565	14,822	3.07	76.9	Stakeholder engagement	National surgical planning: - Conducted baseline situational analysis Targeted interventions: - Collaborations on climate change mitigation and sustainability - Use of solar power	- Access to quality training - Climate change - Antimicrobial resistance - NCDs	- Decentralise health services - Integrate surgical care with prevention - Increase spending to train professionals - Strengthen IPC
Fiji	902,899	5,086	3.82	67.4	Drafting	National surgical planning: - Appointed a national coordinator for safe and affordable surgery - Conducted baseline situational analysis - Stakeholder consultation workshop Targeted interventions: - Significant increase in healthcare worker numbers - Increase in scholarships - Service remodeling to decentralise care	- Geography - Transportation - The COVID-19 pandemic - The rising cost of surgical education	- Strengthen surgical care capacity in first-level hospitals - Remote training - Integrate surgical services with public health and emergency preparedness
Malaysia	32,776,195	11,371	3.83	76.6	Commitment	National surgical planning: - Formed a core working group - Developed a two-phase plan to implement safe and affordable surgery - Assessed surgical needs in 5 out of 13 states - Designated POMR as a key performance indicator Targeted interventions: - Developed a national obstetric database - Anaesthesia task-shifting program	- Poor data quality - Large rural population - Workforce sustainability in Eastern Malaysia	- Conduct site visits in the remaining states - Hold a national forum - Develop an NSOAP - Set achievable standards for workforce training and safety
Nepal	29,674,920	1,223	4.45	70.8	Commitment	- Universal access to emergency care - Subsidised the treatment of many surgical conditions - Anaesthesia task-shifting program - Integrated surgery into national health policy and strategies	- Long wait times for elective surgery - Mountainous geography	- Convene stakeholders - Develop an NSOAP

**Table 2:** Summary of discussion themes by stakeholder

Themes	Population needs		Service design		Infra-structure	Workforce	Information system	Finance	Governance
Stake-holder	Wider context	Disease burden	Integrating surgery with public health	Strengthening surgical care in first-level hospitals		Workforce strengthening	Data collection	Financial risk protection	
Tonga						Remote training	LCoGS indicators		Building political momentum Educating political leaders
Cook Islands	Climate change, COVID-19 pandemic, AMR	NCDs	IPC	Decentralisation of health services		Training Quality supervision Continuous professional development			Developing NSOAP
Fiji	COVID-19 pandemic, climate change, natural disasters		Surgeon involvement in pandemic response Integrating surgical care with emergency response	Decentralisation of surgical care		Health workforce shortage Rising cost of training Scholarships Teleconference			Developing NSOAP
Malaysia						Anaesthetic task-shifting program	National obstetric registry LCoGS indicators POMR		Communication between policymakers and providers
Nepal		Road traffic accidents, cancers				Task-shifting		Subsidising surgical care	Integrating surgery into national policy
WHO WPRO	COVID-19 pandemic	Emergency maternal care, maternal mortality	IPC Waste management	Remote provinces, Outer islands	Access to equipment	Sustainable specialist training			
WHO SEARO	COVID-19 pandemic	Emergency obstetric care, maternal mortality			Blood safety		Monitoring and evaluation of service quality and safety	Increase public health spending for financial risk protection	Multi-year planning Integrating surgical plans into national health plans

### The history and current status of global surgery

Speakers: Nikhil Seth, Executive Director of the United Nations Institute for Training and Research (UNITAR) and UN Assistant Secretary-General.

Salmaan Keshavjee, Director of the Harvard Medical School (HMS) Center for Global Health Delivery.

John Meara, Director of the Program in Global Surgery and Social Change (PGSSC) and Professor of Surgery at HMS.

Elizabeth McLeod, Paediatric Surgeon at the Royal Children’s Hospital, Australia, and RACS Fellow.

#### The evolution of global surgery

Meara provided a historical view of how surgery has risen on the global health agenda. For most of the twentieth century, surgery was not considered part of the global health dialogue. In 2008, two renowned infectious disease experts, Jim Kim and Paul Farmer, described surgery as the ‘neglected stepchild of global health’ [3]. This quote was a powerful metaphor that galvanised the surgical community. This article also debunked false beliefs about surgery: that surgery is too costly, that surgery does not need to be a priority, and that the burden of surgical disease was insignificant. Major developments occurred in 2015,. First, The World Bank published the Disease Control Priorities, 3rd edition series, with the first volume focused on surgery [4]. Second, the Lancet Commission on Global Surgery (LCoGS) published its report outlining six core surgical indicators and calling for national surgical strategic planning [5]. Third, the World Health Assembly (WHA) Resolution 68.15 was passed, calling for strengthening emergency and essential surgical care as an integral component of universal health care (UHC) [6]. Since 2015, there has been rapid growth in research, advocacy, and policy work related to global surgery, including surgical indicators collection and NSOAP development.

#### The contribution of surgical care to UHC and pandemic preparedness

Seth and Keshavjee noted that the value of investing in surgical care has never been more apparent in light of climate change and the COVID-19 pandemic. The COVID-19 pandemic has highlighted the value of robust health systems in providing pandemic preparedness and surge response. Strengthening surgical systems using a broad-based, holistic approach has benefits far beyond surgical care. It will decrease poverty and inequality, promote economic growth, and build strong institutions and partnerships.

McLeod warned that the intersection of multiple present crises, the climate emergency, the COVID-19 pandemic, and the failures of liberal democracy, is likely to further diminish the already limited fiscal space for surgery. Overcoming these problems will require strategic efforts in areas of synergy between surgery and emergency preparedness, such as the Sendai Framework for Disaster Risk Reduction (2015–2030) [7], climate-resilient health infrastructure plans, supply chains, information systems, emergency response teams, and the COVID-19 pandemic response. Meara concurred and urged participants to incorporate surgical care into not only UHC and the Sustainable Development Goals, but also more recent narratives about global health security, pandemic preparedness, and climate change [8]. McLeod invoked the insight of the Lancet Commission for Planetary Health, which suggested that the most likely failures in planetary health will be failures of imagination [9].

#### Progress in the Asia–Pacific region

Meara noted the substantial progress in national surgical planning in the Asia–Pacific region. Pacific Island Countries (PICs) have led the way by collecting, benchmarking, and publishing the first four LCoGS indicators [10]. These indicators have highlighted critical access and workforce issues, with high rates of impoverishing and catastrophic expenditures [10–12]. In 2019, the Pacific Health Ministers championed a Pacific-specific approach to advancing safe and affordable surgery as a critical component of achieving the Healthy Islands vision [13]. PICs are currently developing national surgical strategic plans in the broadest sense by analysing the components and stakeholders in the entire surgical ecosystem.

#### The future of the global surgery movement

McLeod pointed out that since Mahler’s call in 1980 for surgery for all [14], the global surgery movement has evolved through several phases of advocacy. These included cost-effectiveness analyses, economic impact assessments, and estimating the global burden of surgical diseases [15]. More recently, surgery and anaesthesia care have been demonstrated to be necessary for 30% of all hospital admissions [16]. Despite its importance, surgery has still struggled to become a fully recognised and funded aspect of global health. This is partly because surgery requires complex ecosystems with multiple parts to function well. It is also difficult to create narratives that resonate as they did in 2015. Using a framework developed a decade prior to compare the relative success of maternal and child health programs, Shiffman and Spiegel investigated the movement to advance surgical care globally [17]. They reported that the major obstacles to improving surgical care are the lack of leadership, failure to present an organised front, and limited efforts to engage and enlist the voices of patients and civil society. There is also a lack of coherence about how to improve surgical care; for example, failure to achieve consensus on a set of bellwether procedures for paediatric surgery has hindered effective positioning. She highlighted effective partnership as a solution going forward and noted the strong surgery and anaesthesia leadership in the Western Pacific region.

### WHO regional office perspectives

#### WHO Western Pacific Region (WPRO)

*Takeshi Kasai, WHO Regional Director for the Western Pacific,* discussed the Action Framework for Safe and Affordable Surgery in the Western Pacific Region (2021–2030) [11]. The Action Framework, which took about a year to develop, was supported and endorsed by all Member States at the Regional Committee Meeting in 2020 [18]. During the developmental phase, WHO WPRO obtained input from many Member States leaders, who agreed that UHC could not be achieved without safe and affordable surgery. They identified a need to strengthen and redesign their surgical systems through a broader system approach customised to local needs. This approach will allow people living in remote provinces or outer islands to access surgical care, and it will help specialists focus on more specialised surgical procedures. They also identified the need for sustainable specialist training mechanisms and more surgical equipment.

Global efforts to improve surgical care were instrumental in driving WPRO to develop the Action Framework. The LCoGS has been a key partner in catalysing change and defining scalable solutions for improving surgical care quality. WPRO’s approach was to build on, adapt, and customise these global efforts to the regional context. Through discussions with the Member States, including officials in Ministries of Health and healthcare professionals, WPRO realised that this agenda could not only achieve safe and affordable surgery but also strengthen health systems more broadly. Although the COVID-19 pandemic has posed challenges to advancing the Action Framework, it also presents opportunities with significant increases in health services investment. This must be leveraged for surgical system strengthening, which in turn, can be a pathfinder for transforming health systems.

#### WHO South-East Asia Region (SEARO)

*Poonam Singh, WHO Regional Director for South-East Asia,* discussed WHO SEARO’s efforts to strengthen surgical systems. Reducing maternal and under-five mortality is a top priority in the region. Over the last 20 years, the region has substantially reduced maternal mortality, an indicator of equitable access to basic and comprehensive emergency obstetric care. Approximately 15% of women in this region require emergency obstetric care. Substantial progress has been made in strengthening health workforce density and skills, necessary for safe and effective surgical care. Singh was confident that the South-East Asia region would achieve a surgical, anaesthetic, and obstetric provider density of 20 per 100,000 population by 2030. Every country in the region has implemented national blood policies aligned with the WHO strategic framework for blood safety and availability [19].

The region is currently facing significant fiscal challenges due to the COVID-19 pandemic and expected decreases in household incomes. This could lead to an increase in forgone care and out-of-pocket payments. Because of this, WHO has urged all nations in South-East Asia to increase and sustain public health spending and expand financial protection to maintain access to essential services, including surgical care, throughout the COVID-19 response and recovery. Experiences from Thailand and Indonesia have shown that increases in prepayment and public financing can deliver sustained reductions in out-of-pocket payments. Furthermore, India has extended financial protection to more than 500 million people for secondary and tertiary in-patient care packages. WHO will continue to assist all nations in increasing the volume and efficiency of public health expenditures to achieve maximum impact, including surgical system strengthening.

In closing, she had five recommendations for countries (Panel 1).



#### Technical Support for the WHO Regional Action Framework for Safe and Affordable Surgery

**Fig. 1:**
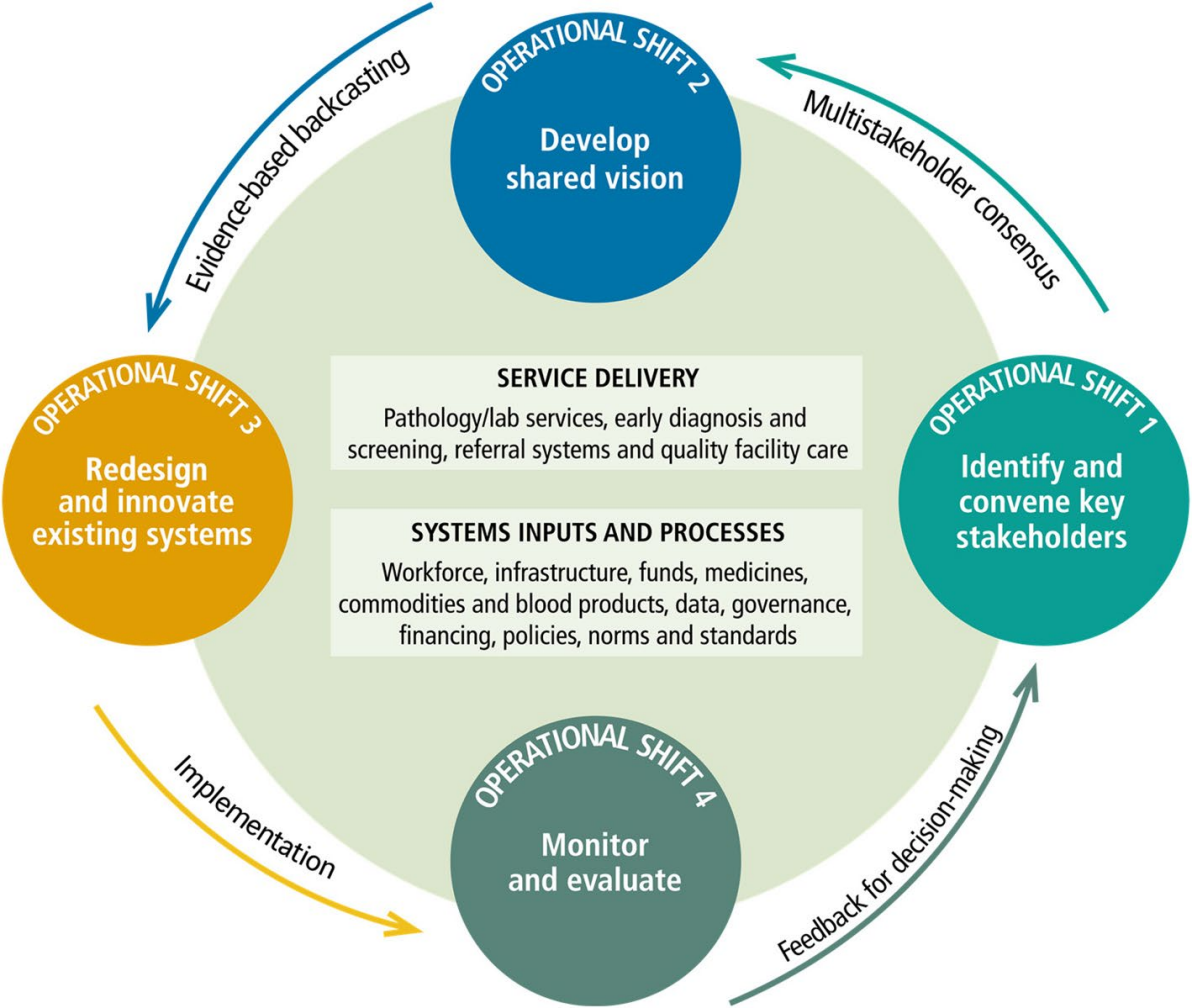
Four operational shifts to achieve safe and affordable surgery [11]

*Howard Sobel, Regional Coordinator for Safe and Affordable Surgery, Quality and Safety, Infection Prevention and Control (IPC), and Maternal and Child Health at the WHO WPRO,* spoke about the priorities and implementation of the Action Framework, which seeks to translate global commitments to the regional level [11]. It comprises four operational shifts and identifies actions that deliver safe and affordable surgery in the short, medium, and long term. The framework calls for establishing a shared vision for surgical care, redesigning health systems to deliver high-quality surgical care, and regular monitoring and recalibration of the shared vision (Fig. 1). Health system redesign requires identifying and addressing critical gaps in health system inputs (e.g., staff and medicines), processes (e.g., quality and safety mechanisms), and essential support services (e.g., supply chains and sterilisation processes) through a unified rather than a siloed approach. These changes often begin at the local level before being replicated and scaled to the national level. For example, in Japan, the implementation of safe and accessible surgical services began at the district and facility levels. Different models must be considered in strengthening surgical care based on local knowledge and priority setting.

Appropriate entry points must be found to secure commitments toward safe and affordable surgery. Furthermore, messaging strategies should be emotionally engaging. For example, a newborn care program became a success when mothers and healthcare workers were emotionally touched by the improved health and apparent happiness of babies in the program. An equally moving and effective approach is needed in surgery. This can generate quick wins that improve the visibility of surgical issues and bring all stakeholders together.

### Ministries of Health Perspectives.

#### Tonga

*Viliami Tangi, Chief Surgeon, former Deputy Prime Minister, and former Minister of Health*, *Kingdom of Tonga.* Tangi remarked that the release of the LCoGS report made him realise the importance of safe and accessible surgery as part of UHC, which he had been previously unaware of during his own career as a politician. The report established key metrics that informed targets for the number of surgeons, anaesthetists, and obstetricians in Tonga. The current landscape is full of opportunities for global collaboration to advance the safe surgery agenda. Still, immediate action is needed to improve surgical care in the region, especially in small developing countries that often require assistance to develop national surgical plans.

#### Malaysia

*Mohamed Yusof bin Abdul Wahab, Head of General Surgical Services for the Malaysia Ministry of Health*. In response to the LCoGS report, the Malaysian government convened a meeting and formed a core team comprising two clinicians and two Ministry of Health officers: the National Heads of Surgical and Anaesthesia Services. They developed a two-phase plan for implementing safe and affordable surgery. Phase one includes implementing essential surgical and anaesthesia services, and phase two adds obstetrics and orthopaedics to the Malaysia national plan. After the meeting, peri-operative mortality rate (POMR) was designated a key performance indicator.^1^

Malaysia consists of two regions, West and East Malaysia, and 13 states. The government funds public healthcare services, while private healthcare is financed by insurance and out-of-pocket expenditure. Public healthcare facilities are located in both rural and urban areas; however, private healthcare facilities are mostly located in urban areas. In each state, a state director runs public health services. State visits were conducted during the planning process, and meetings were held between the core team, the state directors, and the hospital directors. Following these initial meetings, the core team visited the district hospitals to determine health facilities’ needs and challenges and assisted in developing possible interventions. They have completed this process in district hospitals in five states and expect to finish in the remaining states by the end of 2021. A national forum on global surgery scheduled for August 2020 was postponed due to the COVID-19 pandemic.

Malaysia’s surgical indicators are consistent with the LCoGS indicators. Malaysia has collected data for three indicators, except for total surgical volume and impoverishing and catastrophic expenditure. 94% of Malaysians have access to bellwether procedures within two hours^2^ [20]. The Ministry of Health hopes to draft its national plan for surgical care in the near future. This will address Malaysia’s surgical needs and provide much-needed care to the people in the region.

#### Fiji

*Ifereimi Waqainabete, Minister for Health and Medical Services, Fiji,* described Fiji’s LCoGS indicators (Table 3). As a nation of 300 islands, geography and transportation are key issues in Fiji. Although Fiji hopes to significantly improve surgical access within the next four years, it is unlikely to achieve 100% access to bellwether procedures within 2 h.

**Table 3:** Lancet Commission on Global Surgery (LCoGS) indicators in Fiji [5, 10]

Indicator	Target y 2030	2015–6 status in Fiji
*1. Access to timely essential surgery* – the percentage of the population that can access, within 2 h, a facility capable of providing bellwether procedures (Caesarean section, laparotomy, and open fracture management)	80%	67%
*2. Specialist surgical workforce density –* number of surgical, anaesthesia, and obstetric specialists per 100,000 people	20	5.8
*3. Surgical volume*—procedures performed in an operating theatre per 100 000 population per year	5,000	1,490
*4. Peri-operative mortality* – prospective monitoring of all-cause death rate before discharge in patients who have undergone a procedure in an operating theatre	Prospectively monitored	0.83% Not prospectively monitored
*5.* % risk of *catastrophic out-of-pocket expenditure*^*a*^ on surgical care	0%	21%
*6.* % risk of *impoverishment due to out-of-pocket expenditure*^*b*^ on surgical care	0%	24%

^a^Impoverishing expenditure is defined as being pushed into poverty or being pushed further into poverty by out-of-pocket payments [5]

^b^Catastrophic expenditure is defined as direct out-of-pocket payments of greater than 40% of household income net of subsistence needs [5]

Healthcare workforce development is a crucial concern for Fiji. Fiji has been continuously training healthcare personnel for the past six years. There has been a significant increase in the number of doctors, midwives, and nurses employed by the Ministry of Health. Fiji has increased the number of scholarships and student loans for science, technology, engineering, and math (STEM) subjects, including the health sciences. In addition to the two universities in the country, Fiji has a national university where most healthcare professionals in the Pacific region are trained.

In the last 12 months, Fiji has made progress in national surgical planning. The government has appointed a Chief Surgeon as the national coordinator for safe and affordable surgery, obstetrics, and anaesthesia. Fiji will soon develop an NSOAP in collaboration with WHO, RACS, and Harvard PGSSC. The government collaborates with the G4 Alliance to strengthen its development partner network. In response to the COVID-19 pandemic, Fiji suspended the training of surgical doctors to focus on the pandemic response. Surgical trainees were engaged in public health training and disaster relief. They provided surgical services in the highlands after cyclones in Fiji and served patients in field hospitals throughout the past year.

The Fijian government has remodelled its health service provision framework, establishing surgery, anaesthesia, and obstetrics at the forefront of public health. The remodelling focused on integrating clinical services and public health. One key feature is the decentralisation of surgical services. Surgical capacity is being established in smaller health facilities to distribute the workload, improve access, and reduce reliance on referral hospitals. Fiji has three referral hospitals and between 18 and 20 first-level hospitals. In the last 30 years, the government has consolidated surgical capacity into the larger divisional hospitals, leaving the smaller subdivisional hospitals focused on general practice. Twenty subdivisional hospitals in Fiji have been assessed, and strategies have been developed to strengthen their surgical capacity. Surgical capacity cannot be developed separately from general health system capacity. Surgical systems need to be situated within strong health systems, and strong health systems need adequate surgical capacity.

Several threats may interfere with surgical system strengthening in Fiji. First, though the COVID-19 pandemic has raised awareness about the need for healthcare resources, this may not necessarily translate to a reallocation of resources toward NSOAPs. Increasing demand for healthcare workers may deter the implementation of new or existing NSOAPs. The Fijian government aims to increase healthcare resources while moving forward with its NSOAP development. Investment in public health, critical care services, and molecular labs will be insufficient on their own; investment in safe and accessible surgical services is necessary to achieve UHC. Second, the rising cost of surgical training could limit access to educational programs. Some training programs have begun using teleconferencing tools to meet training needs. The surgical apprenticeship model must continue whether training is conducted remotely or in person.

The next generation of surgeons must be proponents of public health to advocate for surgery from within public health. This approach may help future generations of surgeons engage with WHO and UN agencies on surgical strengthening issues. Surgeons make good candidates for public health programs because they are critical thinkers, action-oriented, and results-driven. Surgical training must prepare surgeons and ensure that surgeons remain relevant in the post-COVID environment, both inside and outside the operating theatre.

The Fijian government plans to establish five bellwether hospitals and a WHO Collaborating Center for safe surgery, obstetrics and anaesthesia. The government will continue advocating for safe and affordable surgery, obstetrics, and anaesthesia in coordination with other priorities, such as the COVID-19 pandemic.

#### Cook islands

*Aumea Herman, public health physician, epidemiologist, general practitioner, and former Secretary for Te Marae Ora Cook Islands Ministry of Health*. Improving surgical service requires a well-trained workforce, quality supervision, and continuous professional development. Access to quality training is a significant barrier for the Cook Islands.

Although integrating public health systems with surgical care is critical, preventative care is also important. The Pacific region, along with the entire world, is currently facing four existential threats that will impact surgical services and surgical system development: the COVID-19 pandemic, climate change, antimicrobial resistance (AMR), and non-communicable diseases (NCDs). NCDs are the greatest threat in the Pacific region, and obesity comorbidities severely impact surgical services. More efficient health systems and workforce development are required to solve these challenges. Decentralisation of health services is beneficial, as it allows healthcare professionals to be closer to the people and better understand their needs at the grassroots level. The governments in the Pacific region should increase healthcare spending to train healthcare professionals, including surgeons and public health physicians.

#### Nepal

*Bikash Devkota, Chief of the Quality Standard and Regulation Division at the Ministry of Health and Population*. In Nepal, health policies are developed by the Ministry of Health and implemented by the Department of Health Services and provincial Ministries of Social Development. All citizens of Nepal have access to basic emergency health services. Specialised services are easily accessible and available through health insurance. Surgical services are even more critical now, given the rise in road traffic accidents and cancer.

There are long waiting lists for elective surgery, even simple procedures. Nepal has prioritised and subsidised the treatment of chronic diseases that require surgical care, including renal transplants. Caesarean sections are available free of charge. Surgical care has been integrated into Nepal’s national health policy and other health strategies. A series of discussions on strengthening surgical care has been convened among Nepal’s government and other stakeholders, and it has been recommended that Nepal develop a surgical system strengthening plan. Nepal plans to develop this plan in the near future.

### Panel discussion

#### Building political momentum

Participants discussed strategies to build political momentum for surgical system strengthening. Waqainabete said that a platform-based approach is needed. Surgeons can contribute by participating in non-surgical organisations and advocating for surgical system needs. This is necessary to increase awareness of surgical needs outside the operating theatre. For instance, the COVID-19 pandemic has brought much attention to the public health sector. If surgeons can demonstrate how they contribute to public health needs and UHC, development partners and politicians will take note. Surgical providers should collaborate and network with other organisations and stakeholders so that surgical advocates are present in all areas of health care.

Tangi highlighted the importance of educating politicians, as they are often unaware of surgical systems’ needs. All Ministers of Health around the world want their populations to be healthy and well; however, they may lack knowledge and technical know-how. He shared a personal anecdote wherein a single presentation on surgical care was sufficient to persuade a newly appointed Minister of Health to commit to developing and implementing an NSOAP.

Sobel added that every stakeholder has areas of interest. Advocates must align with stakeholders’ areas of interest to build consensus and secure funding for surgical system strengthening,

#### Engaging obstetrics, gynaecology, and maternal health

Participants asked how to effectively engage obstetrics, gynaecology, and maternal health in surgical system strengthening plans. Sobel said that timely access to emergency care is a significant determinant of maternal mortality. Approximately half of the maternal mortality cases had no identified risk factors but could be averted through emergency interventions, including surgical interventions. Improving access to surgical services, especially obstetric services, may significantly impact maternal health outcomes.

#### Targeting marginalised populations

*Sabrina Juran, Lecturer on Global Health and Social Medicine at Harvard Medical School,* commented that efforts to provide UHC must prioritise reaching and empowering marginalised groups. Marginalised groups often face common challenges, and structural changes could unlock common benefits and opportunities for them. Juran asked about ways to identify vulnerable groups and design interventions accordingly.

Waqainabete noted that the cohort that constitutes marginalised groups varies in each setting. It is crucial to identify marginalised groups in order to address their needs. In Fiji, the government has identified numerous marginalised groups who do not have access to safe and affordable surgery, such as those living in mountainous areas that cannot readily access surgical services.

Wahab discussed the importance of communication between providers and policymakers. Often policymakers’ perceptions of an issue differ from that of providers, as they may be looking at different data and interpreting data differently. Moving forward, it is important to consider what data should be presented to which stakeholders. POMR data can help highlight areas of concern.

#### Simulation-based, remote, and in-country training

*Ram Nataraja, Associate Professor and paediatric surgeon at Monash Children’s Hospital, Australia,* remarked that simulation-based education (SBE) could be used to teach technical skills, teamwork, professional practice, situational awareness, and leadership. He asked whether SBE has been used in the Asia–Pacific region. Waqainabete agreed that SBE is the way forward. SBE could allow training for surgery, obstetrics, and anaesthesia to be conducted on-site at the hospitals where the trainees will work instead of in larger hospitals in major cities. Tangi explained that Tonga is currently focused on maintaining the momentum of its NSOAP development. They have conducted a situational analysis and stakeholder meetings. He agreed that SBE would likely be used in Tonga in the future.

Given the workforce shortages and constraints on training associated with the COVID-19 pandemic, *Basil Leodoro, paediatric and general surgeon, Vanuatu*, asked whether more training could be done in-country with the support of development partners. Tangi noted that COVID-19 travel restrictions, such as quarantine requirements, have made training more difficult. Training has continued throughout the pandemic and is increasingly conducted using online platforms. Trainees in Tonga frequently receive instruction from colleagues in Australia, New Zealand, and the US. *Annette Holian, consultant orthpaedic and trauma surgeon at Monash Children’s Hospital and Councillor at RACS,* explained that RACS is open to supporting any model used by its partners, especially simulation training with split faculty, which offers local and virtual support to enhance trainees’ competencies before they operate on patients.

#### Task-shifting and anaesthesia care strengthening

Participants enquired about task-sharing initiatives in surgical and anaesthesia care. Devkota explained that Nepal trains both specialists and non-specialists anaesthesia providers. More than half of Nepal’s land is mountainous. Anaesthetic assistants, consisting of staff nurses and paramedics, have been trained to provide essential anaesthesia services in first-level hospitals in mountainous regions.

Wahab described a task-shifting program in Malaysia. Most of Malaysia’s population resides in West Malaysia; doctors from West Malaysia provide much of the health services in East Malaysia. This presents a challenge to healthcare workforce sustainability in remote areas of East Malaysia, as doctors from West Malaysia can become homesick. An anaesthesia task-shifting program was developed in Sabah and Sarawak to address this. Nurses were upskilled to perform select anaesthesia procedures according to safety standards. These trained nurses, called ‘general anaesthesia (GA) men’, administer anaesthetics in remote, hard-to-reach areas for various procedures, including Caesarean delivery. The government is working to better manage available resources through training and safety standards so that all patients in need can access care. In some countries, high standards have been set that may be unachievable, reducing the availability of anaesthesia services. Each country must understand its resources and capabilities.

*Wayne Morriss, President-Elect of the World Federation of Societies of Anaesthesiologists**** (****WFSA),* agreed that each country must devise their own workforce solutions. It is challenging to develop high-quality anaesthesia services without leadership from specialist anaesthetists. The WHO-WFSA International Standards for a Safe Practice of Anaesthesia is a WHO-endorsed document that countries can use as a guide for developing anaesthesia services [21]. It offers a graded approach to anaesthesia service development, which can help prevent countries from setting unachievable standards.

#### The impact of climate change on surgical service delivery

Leodoro commented on the potentially significant impact of climate change on access to surgery. This was overlooked in the LCoGS and the global surgery community must examine this issue now. *Craig McClain, paediatric anaesthetist at Boston Children's Hospital, USA,* asked what steps could be taken to mitigate climate change and promote sustainability while developing surgical systems.

Waqainabete explained that Fiji is subject to natural disasters and cyclones, and Fijians are determined to make their infrastructure and facilities more resilient to withstand future disasters. Fiji has embedded emergency services, public health services, and clinical health services for emergency response. Temporary hospitals are set up to respond to natural disasters. Workers are dispatched to make local hospitals functional as quickly as possible.

Herman discussed the need for robust IPC systems in PICs. There is limited access to clean water in many low-resource areas. The ongoing COVID-19 pandemic has underscored the importance of IPC. Some PICs have made progress in energy production sustainability; for example, Tuvalu and the Cook Islands have solar power. Rising sea levels are intruding into water storage spaces throughout the Asia–Pacific region. Due to the impact of climate change, PICs may face water shortages. These countries must acquire the ability to produce water through desalination or other methods to maintain access to clean water, which is critical in providing safe surgical services. Additionally, all health professionals need access to clean water for hand-washing to prevent nosocomial infections. Currently, PICs and their development partners are collaborating on many projects on climate change mitigation and health system sustainability.

Sobel described a study conducted in delivery rooms and post-natal wards of 147 hospitals across East Asia and the Pacific regions. It found that while most wards had access to sinks and running water, fewer had access to soap. Only 10 to 40% of wards had access to hygienic hand drying methods [22]. In many settings, simply increasing the availability of hand-washing and drying facilities could significantly improve IPC. Sobel also raised the issue of pollution, explaining that health facilities contribute to air pollution and environmental degradation when they fail to segregate or treat biohazardous waste. Health facilities contribute between 8 and 11% of overall pollution, depending on the indicator assessed. He emphasised the importance of proper hospital waste disposal to curb the impacts of climate change.

#### Identifying priorities for surgical system strengthening

*William May, Dean of the College of Medicine, Nursing, and Health Sciences at Fiji National University,* noted that safe surgery is cross-cutting, impacting policy, practice, people, facilities, and equipment. He asked how countries can identify priorities for improving surgical systems. Watters said that the first step is creating a national plan for improving surgical care that includes measuring progress using key performance indicators, such as the LCoGS indicators. Surgical plans should be integrated into national health plans, which typically do not specifically address surgical care. What is not measured goes unmanaged, said May. Moreover, improving access to bellwether-capable hospitals is crucial to surgical system improvement.

#### Engaging nurses, trainees, and students

Webinar attendees expressed the enthusiasm of nurses, trainees, and students to advocate for and support surgical system strengthening. Participants acknowledged that nurses can often be inadequately represented in global surgery forums. Moving forward, a broad-based, inclusive movement should be built. Participants encouraged surgical residents to join professional associations in their respective countries and regions. They suggested that medical students could contribute by developing a passion for public health and health system thinking.

#### Impact of the COVID-19 pandemic on surgical care delivery and research

Participants discussed strategies to advance access to surgical care during the COVID-19 pandemic. They agreed that though broader factors, such as governance and public health policies, play a critical role, health systems with robust surgical care capacity typically fared better throughout the pandemic. This is because surgical system strengthening focuses on holistic health system improvement rather than individual diseases or vertical programs. Surgical system strengthening could lead to more robust health systems and better pandemic preparedness.

Participants discussed the impact of the COVID-19 pandemic on global surgery research initiatives and how collaborators can help improve surgical research capacity. It was suggested that researchers from high-income countries (HICs) create partnerships and collaborations with researchers in low- and middle-income countries (LMICs) to support them in research capacity building. This research must not be conducted in a predatory manner; the benefits and credit for research should not be attained primarily by HIC researchers and institutions.

## Conclusion

During this session, Ministries of Health and WHO Regional Offices in the Asia–Pacific region discussed the current status, challenges, and opportunities to improve surgical, obstetric, and anaesthesia care in the Asia–Pacific region. Participants highlighted the substantial progress made to date, including developing NSOAPs, workforce strengthening, reducing maternal mortality, improving blood access, and establishing data systems among other areas. The COVID-19 pandemic and climate change pose significant challenges to strengthening surgical care in the Asia–Pacific region. Participants called for reimagining surgical education and care delivery by integrating it closely with public health, preventive care, and preparedness to emergencies, including pandemics and natural disasters. Addressing these regional challenges require forming collaborations beyond the health sector, for example, with environmental management. Countries shared challenges in providing care to rural, hard-to-reach populations and proposed solutions, such as task-shifting, outreach, and strengthening surgical capacity in first-level hospitals.

Going forward, there is an ongoing need to build political momentum through using data and evidence. The movement to advance global surgery must not only be cohesive but also broad-based and inclusive of the voice of all specialties, students, and most importantly service users. Future policy discussions in global surgery should be centred around patient journeys and patient experiences. The COVID-19 pandemic has provided an opportunity for increased healthcare investment and remote, simulation-based education, which could be leveraged into the future.

The strength of this session is the number of high-level country and regional representatives involved. Since this session, the countries represented have already made further progress in national surgical planning, with the Pacific Island Countries being further in the NSOAP development stage and Nepal and Malaysia moving from the commitment to the development stage. Due to the virtual format of this session, there was limited scope to build regional collaboration. Platforms provided by regional organisations, such as the WHO regional offices, should be established to facilitate technical sharing and the generation of regional-level solutions.

## Funding 

This meeting and publication were funded by the Harvard Medical School Center for Global Health Delivery. The funders had no role in preparation of the meeting content or meeting report.

## Supplementary Information

**Additional file 1:** **About this supplement. **This article has been published as part of *BMC Proceedings Volume 17 Supplement 5, 2023: Strategic Planning to Improve Surgical, Obstetric, Anaesthesia, and Trauma Care in the Asia-Pacific Region.* The full contents of the supplement are available online at https://bmcproc.biomedcentral.com/articles/supplements/volume-17-supplement-5.

## Data Availability

N/A.

## Abbreviations

CHE: Current health expenditure
GDP: Gross domestic product
HICs: High-income countries
HMS: Harvard Medical School
IPC: Infection prevention and control
LCoGS: Lancet Commission on Global Surgery
LMICs: Low- and middle-income countries
NCD: Noncommunicable disease
NSOAP: National Surgical, Obstetric, and Anaesthesia Plan
PGSSC: Program in Global Surgery and Social Change
PIC: Pacific Island Country
POMR: Peri-operative mortality rate
RACS: Royal Australasian College of Surgeons
STEM: Science, technology, engineering, and math
UHC: Universal health coverage
UNITAR: United Nations Institute for Training and Research
WFSA: World Federation of Societies of Anaesthesiologists
WHA: World Health Assembly
WHO: World Health Organization
WHO CC: World Health Organization Collaborating Center
WHO SEARO: World Health Organization South-East Asia Region
WHO WPRO: World Health Organization Western Pacific Region

## Footnotes

1 The peri-operative mortality rate is defined by the LCoGS as ‘the all-cause death rate before discharge in patients who have undergone a procedure in an operating theatre, divided by the total number of procedures, presented as a percentage’.[5]

2 Bellwether procedures include laparotomy, caesarean delivery, and treatment of open fractures. These procedures serve as bellwethers of a health system’s level of complexity and capacity to provide most other emergency and essential surgical procedures.[5]

## References

[1] World Bank World Bank Open Data. In: World Bank Open Data. https://data.worldbank.org/. Accessed 29 Oct 2022.

[2] Ora Te Marae (2020). National Health Information Bulletin 2019–2020.

[3] Farmer PE, Kim JY (2008). Surgery and global health: a view from beyond the OR. World J Surg.

[4] Debas HT, Donkor P, Gawande A, Jamison DT, Kruk ME, Mock CN (eds) (2015) Essential Surgery: Disease Control Priorities, Third Edition (Volume 1). World Bank, Washington . 26740991

[5] Meara JG, Leather AJM, Hagander L (2015). Global Surgery 2030: evidence and solutions for achieving health, welfare, and economic development. Lancet.

[6] WHO (2015). WHA 68.15: strengthening emergency and essential surgical care and anaesthesia as a component of universal health coverage.

[7] UNDRR (2015) Sendai Framework for Disaster Risk Reduction 2015–2030. United Nations Office for Disaster Risk Reduction.

[8] Roa L, Jumbam DT, Makasa E, Meara JG (2019). Global surgery and the sustainable development goals. BJS (British Journal of Surgery).

[9] Whitmee S, Haines A, Beyrer C (2015). Safeguarding human health in the Anthropocene epoch: report of The Rockefeller Foundation-Lancet Commission on planetary health. Lancet.

[10] Guest GD, McLeod E, Perry WRG (2017). Collecting data for global surgical indicators: a collaborative approach in the Pacific Region. BMJ Glob Health.

[11] World Health Organization Regional Office for the Western Pacific (2021) Action framework for safe and affordable surgery in the Western Pacific Region: 2021–2030. Manila.

[12] Shrime MG, Dare AJ, Alkire BC, O’Neill K, Meara JG (2015). Catastrophic expenditure to pay for surgery worldwide: a modelling study. Lancet Glob Health.

[13] WHO (2019). Outcomes of the Thirteenth Pacific Health Ministers Meeting.

[14] Mahler H (1980). “Surgery and health for all” - Address by Dr H. Mahler Director-General of the World Health Organization to the XXIII biennial world congress of the International College of Surgeons.

[15] Rose J, Weiser TG, Hider P, Wilson L, Gruen RL, Bickler SW (2015). Estimated need for surgery worldwide based on prevalence of diseases: a modelling strategy for the WHO Global Health Estimate. Lancet Glob Health.

[16] Fehlberg T, Rose J, Guest GD, Watters D (2019). The surgical burden of disease and perioperative mortality in patients admitted to hospitals in Victoria, Australia: a population-level observational study. BMJ Open.

[17] Shawar YR, Shiffman J, Spiegel DA (2015). Generation of political priority for global surgery: a qualitative policy analysis. Lancet Glob Health.

[18] World health Organization Regional Committee for the Western Pacific (2020). World Health Organization Regional Committee for the Western Pacific Resolution on Safe and Affordable Surgery.

[19] World Health Organization (2020). Blood safety and availability.

[20] Hoh SM, Wahab MYA, Hisham AN, Guest GD, Watters DAK (2021). Mapping timely access to emergency and essential surgical services: The Malaysian experience. ANZ J Surg.

[21] Gelb AW, Morriss WW, Johnson W (2018). World Health Organization-World Federation of Societies of Anaesthesiologists (WHO-WFSA) International Standards for a Safe Practice of Anesthesia. Anesth Analg.

[22] Mannava P, Murray J, Kim R, Sobel H (2019) Status of water, sanitation and hygiene services for childbirth and newborn care in seven countries in East Asia and the Pacific. 10.7189/jogh.09.020430. 10.7189/jogh.09.020430 PMC6925970 31893033

