# Supplementary material for: The current status of surgical care in the Asia–Pacific region and opportunities for improvement: proceedings
Source: BMC Proc. 2023 Jul 25;17(Suppl 5):12. doi: 10.1186/s12919-023-00255-0 (PMC10367230; doi:10.1186/s12919-023-00255-0)
Supplement: Supplementary file 1 — Additional file 1: About this supplement. This article has been published as part of BMC Proceedings Volume 17 Supplement 5, 2023: Strategic Planning to Improve Surgical, Obstetric, Anaesthesia, and Trauma Care in the Asia-Pacific Region. The full contents of the supplement are available online at https://bmcproc.biomedcentral.com/articles/supplements/volume-17-supplement-5. [file 12919_2023_255_MOESM1_ESM.docx]

**About this supplement**

This article has been published as part of *BMC Proceedings Volume 17 Supplement 5, 2023: Strategic Planning to Improve Surgical, Obstetric, Anaesthesia, and Trauma Care in the Asia-Pacific Region.* The full contents of the supplement are available online at https://bmcproc.biomedcentral.com/articles/supplements/volume-17-supplement-5.
